# Assessment of Communication Strategies for Mitigating COVID-19 Vaccine-Specific Hesitancy in Canada

**DOI:** 10.1001/jamanetworkopen.2021.26635

**Published:** 2021-09-30

**Authors:** Eric Merkley, Peter John Loewen

**Affiliations:** 1 Department of Political Science, University of Toronto, Toronto, Ontario, Canada; 2 Munk School of Global Affairs and Public Policy, University of Toronto, Toronto, Ontario, Canada

## Abstract

**Importance:**

Ensuring widespread uptake of available COVID-19 vaccinations, each with different safety and efficacy profiles, is essential to combating the unfolding pandemic.

**Objective:**

To test communication interventions that may encourage the uptake of less-preferred vaccines.

**Design, Setting, and Participants:**

This online survey was conducted from March 24 to 30, 2021, using a nonprobability convenience sample of Canadian citizens aged 18 years or older, with quota sampling to match 2016 Canadian Census benchmarks on age, gender, region, and language. Respondents completed a 2-by-2-by-2 factorial experiment with random assignment of brand (AstraZeneca or Johnson & Johnson), information about the vaccine’s effectiveness against symptomatic infection (yes or no), and information about the vaccine’s effectiveness at preventing death from COVID-19 (yes or no) before being asked about their willingness to receive their assigned vaccine and their beliefs about its effectiveness.

**Exposures:**

Respondents were randomly assigned a vaccine brand (AstraZeneca or Johnson & Johnson) and information about the vaccine’s effectiveness against symptomatic COVID-19 infection (yes or no) and at preventing death from COVID-19 (yes or no).

**Main Outcomes and Measures:**

Respondents’ self-reported likelihood of taking their assigned vaccine if offered (response categories: very likely, somewhat likely, not very likely, or not at all likely, scaled 0-1) and their beliefs about their assigned vaccine’s effectiveness (response categories: very effective, somewhat effective, not very effective, or not at all effective, scaled 0-1) were measured.

**Results:**

A total of 2556 Canadian adults responded to the survey (median [IQR] age, 50 [34-63] years; 1339 women [52%]). The self-reported likelihood of taking an assigned AstraZeneca or Johnson & Johnson vaccine was higher for respondents given information about their assigned vaccine’s effectiveness at preventing death from COVID-19 (*b*, 0.04; 95% CI, 0.01 to 0.06) and lower among those given information about its overall effectiveness at preventing symptomatic transmission (*b*, −0.03; 95% CI, −0.05 to 0.00), compared with those who were not given the information. Perceived effectiveness was also higher among those given information about their assigned vaccine’s effectiveness at preventing death from COVID-19 (*b*, 0.03; 95% CI, 0.01 to 0.05) and lower among those given information about their assigned vaccine’s overall efficacy at preventing symptomatic infection (*b*, −0.05; 95% CI, −0.08 to −0.03), compared with those who were not given this information. The interaction between these treatments was neither substantively nor statistically significant.

**Conclusions and Relevance:**

These findings suggest that providing information on the effectiveness of less-preferred vaccines at preventing death from COVID-19 is associated with more confidence in their effectiveness and less vaccine-specific hesitancy. These results can inform public health communication strategies to reduce hesitancy toward specific COVID-19 vaccines.

## Key Points

**Question:**

Are there communication strategies that can reduce COVID-19 vaccine-specific hesitancy?

**Findings:**

In a between-participants survey study of adult Canadian citizens, individuals who were provided information on the death prevention potential of less-preferred vaccines, such as AstraZeneca and Johnson & Johnson, reported more confidence in their effectiveness and a higher likelihood of taking these vaccines if offered compared with those who did not receive this information. Information on the overall effectiveness of these vaccines at preventing symptomatic COVID-19 showed the opposite result.

**Meaning:**

These findings suggest that communication strategies that focus on the death prevention potential of less-preferred COVID-19 vaccines have the potential to improve their uptake, whereas focusing on such metrics as their comparatively less impressive overall effectiveness at preventing symptomatic COVID-19 could undermine these efforts.

## Teaser

This survey study examines communication interventions that may encourage the uptake of less-preferred vaccines against COVID-19.

## Introduction

Mass vaccination campaigns are rapidly proceeding globally. These campaigns make use of vaccines with different characteristics, such as their country of origin, number of required doses, underlying technology (eg, mRNA), and their levels of efficacy and safety. Containing the COVID-19 pandemic will require vaccinating at least 70% of US individuals^1^ and billions more globally. It will also likely require follow-up or booster vaccinations. Understanding the sources of hesitancy and identifying correctives is, thus, vitally important for global public health.

Important research identifies sources of COVID-19 vaccine hesitancy. Resistance appears to be higher among people with low trust in expertise^2^ and, in the US, among Republican party identifiers.^3^ Vaccine hesitancy is also higher among racial and ethnic minority groups, women, those with more skepticism about childhood vaccines, and those skeptical of the severity of the COVID-19 pandemic.^2^ This work^2^ aligns with findings on which groups of citizens are more likely to comply with public health directives on mask usage and social distancing^4,5,6,7^ and broader findings on vaccine hesitancy outside of the COVID-19 context.^8,9,10,11,12,13^

Those previous works emphasize attitudinal and demographic correlates, but it is also possible that the characteristics of vaccines matter for vaccine hesitancy. Some research^14,15,16^ has shown that although people are unresponsive to the technology of the vaccine or the dosing regimen, they are more likely to take vaccine candidates that are domestically produced and with higher levels of safety and efficacy.

The previous studies^14,15,16^ tested people’s responsiveness to the characteristics of hypothetical vaccine candidates. However, the implications may well extend to the real world. Initial trials of AstraZeneca and Johnson & Johnson revealed less efficacy in preventing symptomatic COVID-19 compared with vaccines by Pfizer and Moderna. At the same time, these vaccines have relatively equal efficacy in preventing death and hospitalization from COVID-19,^17,18^ a factor that may be lost in the noise of competing public health directives for vaccine usage. Furthermore, regulators in Europe and North America have raised concerns about the association between AstraZeneca and, more recently, Johnson & Johnson and serious blood clots, which has been widely covered in the news media, possibly further undermining confidence.

Considering the importance of the Johnson & Johnson and AstraZeneca COVID-19 vaccines to global supply, identifying ways to mitigate hesitancy toward these specific vaccines is vitally important. Canada serves as a useful test case for these dynamics. The national regulator, Health Canada, has approved 4 COVID-19 vaccines (Moderna, Pfizer, AstraZeneca, and Johnson & Johnson), each with different usage guidelines, levels of availability, and safety and efficacy profiles. In this study, we examine whether providing information on the effectiveness of the AstraZeneca and Johnson & Johnson vaccines at preventing death from COVID-19 increases people’s confidence in their effectiveness and reduces their hesitancy toward these vaccines. We also explore whether this information can mitigate possible negative consequences that arise from providing information on the comparatively less-impressive record of these vaccines at preventing symptomatic COVID-19 infection.

## Methods

This survey study was approved by the Social Science, Humanities, and Education Research Ethics Board at the University of Toronto. It was fielded from March 24 to 30, 2021, on an online nonprobability sample of adult Canadian citizens using the survey respondent panel provider Dynata, who use quota-based sampling to approximate nationally representative samples. In this case, quotas were set on age (ie, age 18-34, 35-54, and ≥55 years), gender (ie, male and female), region (ie, Atlantic, Quebec, Ontario, and West), and language (ie, English and French) to match population benchmarks in the 2016 Canadian census. Respondents were provided a consent form at the start of the survey and indicated their consent by proceeding to complete the survey. They could withdraw their consent at any time by closing their browser. The terms of compensation provided by Dynata to members of their panel and those who participated in our survey are proprietary. This study follows the American Association for Public Opinion Research (AAPOR) reporting guideline.

Although probability samples are needed for accurate estimates of the population, nonprobability samples perform well at studying associations between variables or for the estimation of sample average treatment effects, which is our focus here.^19^ Data are not weighted in the analyses that follow.

The study was a 2-by-2-by-2 factorial experiment. Respondents were randomly assigned into brand conditions using the Qualtrics survey platform (ie, AstraZeneca or Johnson & Johnson) and into conditions where they either received or did not receive information on the death prevention effectiveness of their assigned vaccine and its overall effectiveness at preventing symptomatic COVID-19. eTable 1 in the Supplement shows that random assignment was successful.

All respondents were given the following prime about their potential COVID-19 vaccine where the brand was randomly assigned: “Health Canada has recently approved the [AstraZeneca/Johnson & Johnson] vaccine*.*” Individuals in the control condition only received the preceding prime. Another group received additional information on the overall effectiveness of their randomly assigned vaccine at preventing cases of symptomatic COVID-19 where the number depended on whether they were assigned AstraZeneca (62%) or Johnson & Johnson (72%): “Studies have shown that the vaccine is [62%/72%] effective at preventing symptomatic COVID-19.” Another group instead received information that their assigned vaccine is virtually 100% effective at preventing death from COVID-19: “Studies have shown that the vaccine is nearly 100% effective at preventing death from COVID-19.” A final group received both pieces of information about their assigned vaccine.

All respondents were asked “How likely would you be to take this vaccine if offered to you?” (response categories: very likely, somewhat likely, not very likely, or not at all likely), and “How would you rate the effectiveness of the vaccine? (response categories: very effective, somewhat effective, not very effective, or not at all effective). The outcomes were rescaled from 0 to 1 so that higher values mean a higher likelihood of taking the vaccine and more confidence in its effectiveness.

We conducted a preregistered pilot of this study, fielded from March 8 to 18, 2021, using identical data collection procedures. The only difference in this design was the absence of brand information in the control condition. That pilot and its hypothesis testing can be found in the eAppendix and eFigure 1 in the Supplement.

### Statistical Analysis

We preregistered expectations that vaccination intention and perceived effectiveness should be higher in the death prevention information conditions and lower in the overall efficacy conditions than when respondents were not given this information. We estimated a pair of models using ordinary least squares regression where we regressed intention and perceived effectiveness on binary variables for our overall efficacy and death prevention conditions, as follows: intention / effectiveness = α + β_1_death_info + β_2_efficacy_info + ε. It is also possible that providing one type of information attenuates the association of the other, so we then estimated another pair of models where we interacted our treatment conditions. We break our results down by brand in eFigure 2 in the Supplement: intention / effectiveness = α + β_1_death_info + β_2_efficacy_info + β_3_death_info × efficacy_info + ε.

In addition to our preregistered analysis, we conducted exploratory subgroup analyses to assess whether the association between the treatments and vaccination intention varied by demographic groups. Respondents self-identified their education, income, age, location of residence, gender, and race and ethnicity as part of the survey. These demographic variables were assessed to determine the associations between the treatment and vaccination intentions among different groups of respondents, which has implications for whom we should be targeting with persuasive messages regarding less-preferred vaccines. We estimated a series of models where we interacted both treatments with categorical variables for bachelor’s degree (designated as 1), middle income and high income (reference, low income), age 35 to 54 years old or 55 years and older (reference, 18-34 years), residence in a large city (designated as 1), visible membership in a racial or ethnic minority group (designated as 1), and gender (1 = female, 0 = male): intention = α + β_1_death_info + β_2_efficacy_info + β_3_moderator + β_4_death_info × moderator + β_5_efficacy_info × moderator + ε. Significance was set at *P* < .05 and was determined using 2-sided *t* tests. Data analysis was performed using Stata statistical software version 16 (StataCorp).

## Results

**Figure 1.**
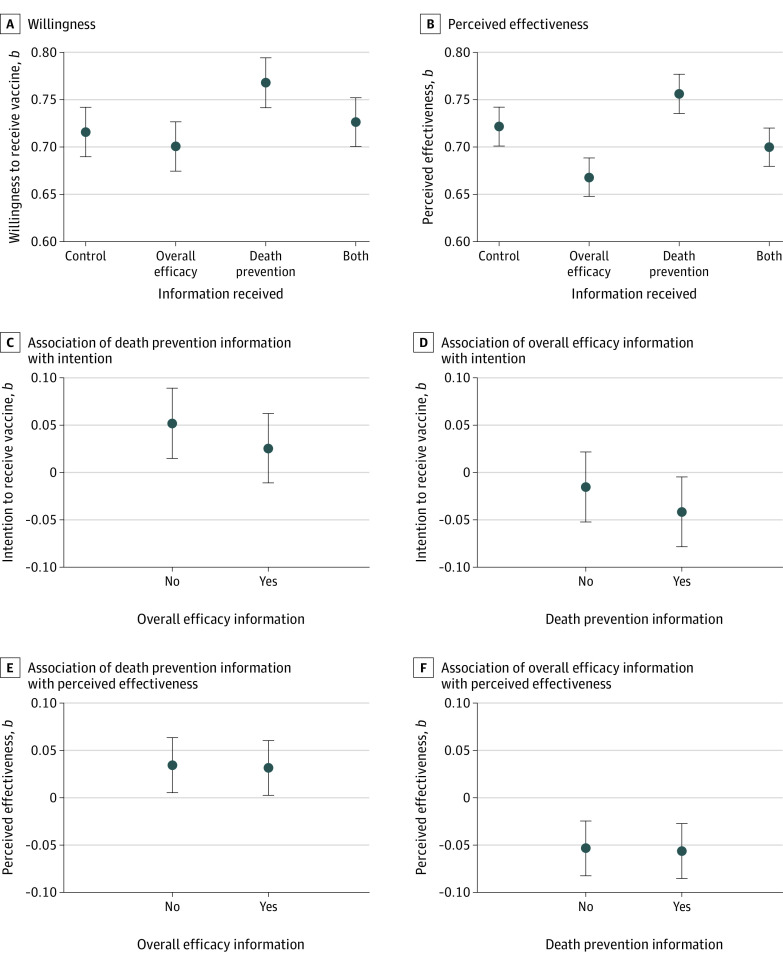
Vaccine Intention and Perceived Effectiveness Across Treatment Conditions Graphs show treatment group means of vaccine willingness (A) and perceived effectiveness (B), and the coefficient estimates (*b*) for the association between death prevention information and intention for respondents who did or did not get the overall efficacy information (C), overall efficacy information and intention for respondents who did or did not get the death prevention information (D), death prevention information and perceived effectiveness for respondents who did or did not get the overall efficacy information (E), overall efficacy information and perceived effectiveness for respondents who did or did not get the death prevention information (F). Error bars denote 95% CIs. Regression estimates are found in eTable 3 in the Supplement. The survey was fielded from March 24 to 30, 2021.

The 2556 survey respondents had a median (IQR) age of 50 (34-63) years, and 1339 (52%) were women. A full comparison between the demographic characteristics of the sample and population benchmarks can be found in eTable 2 in the Supplement. The group means are provided in Figure 1. We found confirmation that intention (*b*, 0.04; 95% CI, 0.01 to 0.06; *P* = .004) and perceived vaccine effectiveness were higher for respondents given the death prevention information than for those who were not (*b*, 0.03; 95% CI, 0.01 to 0.05; *P* = .002). Intention (*b*, −0.03; 95% CI, −0.05 to −0.00; *P* = .03) and perceived effectiveness were also lower for those given information on the overall efficacy of their assigned vaccine compared with those who were not (*b*, −0.05; 95% CI, −0.08 to −0.03; *P* < .001).

We failed to find evidence of an interaction between the treatments—that is, providing one type of information did not appear to attenuate the association between receiving the other type and the outcomes, as shown in Figure 1. The associations between the death prevention information and the outcomes were the same whether or not a respondent received information about overall efficacy. Likewise, the negative association between overall efficacy information and outcomes remained whether or not a respondent received information about death prevention. Rather than overall efficacy and death prevention information interacting, their associations with the outcomes were additive. The model estimates are found in eTable 3 in the Supplement. The outcomes were not continuous, so for robustness we estimated our models using ordinal logistic regression. These estimates are provided in eTable 4 in the Supplement, and the results for the interaction model are graphically presented in eFigure 3 in the Supplement.

**Figure 2.**
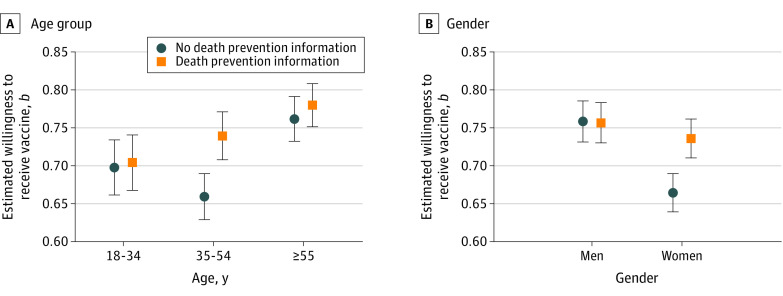
Estimated Vaccine Intention for Those Given the Death Prevention Information and Those Who Were Not by Age Group and Gender Graphs show mean coefficient estimates (*b*) for age group (A) and gender (B). Error bars denote 95% CIs. Regression estimates are found in Table 1 and Table 2. Survey was fielded from March 24 to 30, 2021.

Our exploratory subgroup analyses identified 2 important moderators of the death prevention treatment on intention: age and gender. The full estimates for our subgroup analyses are provided in Table 1 and Table 2. We illustrate the results graphically with linear estimates in Figure 2. Among respondents aged 35 to 54 years, intention was 0.08 point higher on a 0 to 1 scale for those given the death prevention information compared with those who were not (95% CI, 0.04 to 0.12; *P* < .001), whereas there was no significant difference between the conditions among those younger than 35 years (*b*, 0.00; 95% CI, −0.05 to 0.06; *P* = .81) and those older than 54 years (*b*, 0.02; 95% CI, −0.02 to 0.06; *P* = .39). The result is that the gap in intention between middle-aged and older respondents was markedly smaller in the treatment condition compared with the control, as shown in Figure 2.

**Table 1.** Subgroup Estimates for Education, Income, and Age, Ordinary Least Squares Estimation

Variables	Degree^a^		Income^a^		Age^a^	
	*b* (95% CI)	*P* value	*b* (95% CI)	*P* value	*b* (95% CI)	*P* value
Death prevention	0.05 (0.02 to 0.08)	.004	0.03 (−0.01 to 0.07)	.15	0.00 (−0.05 to 0.06)	.86
Overall efficacy	−0.03 (−0.06 to 0.00)	.07	−0.06 (−0.10 to −0.02)	.007	0.00 (−0.05 to 0.05)	.91
Degree	0.07 (0.02 to 0.11)	.006	NA	NA	NA	NA
Degree × death prevention	−0.03 (−0.09 to 0.02)	.28	NA	NA	NA	NA
Degree × overall efficacy	−0.00 (−0.06 to 0.05)	.96	NA	NA	NA	NA
Middle income	NA	NA	0.01 (−0.05 to 0.06)	.85	NA	NA
High income	NA	NA	0.08 (0.02 to 0.14)	.01	NA	NA
Middle income × death prevention	NA	NA	0.01 (−0.06 to 0.07)	.85	NA	NA
High income × death prevention	NA	NA	−0.02 (−0.09 to 0.05)	.62	NA	NA
Middle income × overall efficacy	NA	NA	0.03 (−0.03 to 0.09)	.36	NA	NA
High income × overall efficacy	NA	NA	0.06 (−0.01 to 0.13)	.12	NA	NA
Age 34-54 y	NA	NA	NA	NA	−0.02 (−0.08 to 0.04)	.54
Age ≥55 y	NA	NA	NA	NA	0.09 (0.03 to 0.14)	.003
Age 35-54 y × death prevention	NA	NA	NA	NA	0.08 (0.01 to 0.14)	.03
Age ≥55 y × death prevention	NA	NA	NA	NA	0.02 (−0.05 to 0.08)	.65
Age 35-54 y × overall efficacy	NA	NA	NA	NA	−0.04 (−0.11 to 0.03)	.25
Age ≥55 y × overall efficacy	NA	NA	NA	NA	−0.05 (−0.11 to 0.02)	.17
Constant, mean (SD)	0.70 (0.68)		0.72 (0.68)		0.70 (0.65)	
Participants, No.	2540		2329		2556	
*R* ^2^	0.01		0.02		0.02	

Abbreviations: *b*, coefficient estimate; NA, not applicable.

^a^ Reference categories are no degree, low income, and age 18 to 34 years.

**Table 2.** Subgroup Estimates for City Resident, Racial or Ethnic Minority Group Membership, and Gender, Ordinary Least Squares Estimation

Variables	City resident^a^		Visibly member of racial or ethnic minority group^a^		Gender^a^	
	*b* (95% CI)	*P* value	*b* (95% CI)	*P* value	*b* (95% CI)	*P* value
Death prevention	0.04 (0.01 to 0.08)	.01	0.04 (0.02 to 0.07)	.003	−0.00 (−0.04 to 0.04)	.93
Overall efficacy	−0.03 (−0.07 to −0.00)	.046	−0.03 (−0.05 to 0.00)	.08	−0.01 (−0.05 to 0.02)	.44
Large city resident	0.03 (−0.02 to 0.08)	.21	NA	NA	NA	NA
City × death prevention	−0.01 (−0.07 to 0.04)	.59	NA	NA	NA	NA
City × overall efficacy	0.01 (−0.04 to 0.07)	.62	NA	NA	NA	NA
Visibly member of racial or ethnic minority group	NA	NA	0.00 (−0.05 to 0.06)	.91	NA	NA
Racial or ethnic minority group × death prevention	NA	NA	−0.03 (−0.10 to 0.04)	.38	NA	NA
Racial or ethnic minority group × overall efficacy	NA	NA	−0.02 (−0.09 to 0.05)	.59	NA	NA
Female	NA	NA	NA	NA	−0.08 (−0.13 to −0.04)	<.001
Female × death prevention	NA	NA	NA	NA	0.07 (0.02 to 0.13)	.006
Female × overall efficacy	NA	NA	NA	NA	−0.02 (−0.08 to 0.03)	.35
Constant, mean (SD)	0.71 (0.68)		0.72 (0.70)		0.77 (0.73)	
Participants, No.	2556		2556		2542	
*R* ^2^	0.01		0.01		0.02	

Abbreviations: *b*, coefficient estimate; NA, not applicable.

^a^ Reference categories are not resident of a large city, not visibly a member of a racial or ethnic minority group, and male gender.

Among women, intention was also 0.07 point higher for those given the death prevention information compared with those who were not (95% CI, 0.04 to 0.11; *P* < .001), whereas there was no significant difference among men (*b*, −0.00; 95% CI, −0.04 to 0.04; *P* = .93). The result is that the gap in intention between men and women is considerably smaller in the treatment condition, as shown in Figure 2. We found no significant subgroup differences in the associations between the provision of overall efficacy information treatment and vaccination intention.

It is worth noting that in Canada, AstraZeneca’s vaccine was targeted first toward people aged 50 to 55 years, then those aged 40 to 55 years, and finally those aged 30 to 55 years. It is this group for whom AstraZeneca was most relevant. We analyzed our results for age and gender by brand and found that the result for age (but not gender) was found exclusively for those in the AstraZeneca condition. These results are presented graphically in eFigure 4 in the Supplement.

## Discussion

In a context of rapidly changing and sometimes contradictory advisories on the use of COVID-19 vaccines, this survey study found that individuals were not favorably disposed toward those produced by AstraZeneca and Johnson & Johnson. This was true in a national context (Canada), where several vaccines have been approved but are not equally available to all citizens.

It is an open question whether such brand preferences would exist absent the potential of receiving another vaccine. It is also an open question, given brand preferences, whether countries may actually slow down vaccination rates by procuring multiple types of vaccine, only some of which citizens will be willing to take if the potential of waiting for another vaccine exists. Nonetheless, given the importance of the Johnson & Johnson, and especially AstraZeneca, vaccines to global supply, mitigating hesitancy toward these vaccines is vitally important.

We found that providing information to respondents about the effectiveness of AstraZeneca or Johnson & Johnson at preventing death from COVID-19 was associated with more confident beliefs in the effectiveness of their assigned vaccine and more willingness to take it if offered. This appeared to be true particularly among middle-aged respondents for whom AstraZeneca was targeted, as well as women. On the other hand, providing individuals with information on the comparatively less-impressive ability of their assigned vaccine to prevent any symptomatic COVID-19 infection was associated with less confidence in its effectiveness and less willingness to take it if offered. Unfortunately, information about the impressive record of AstraZeneca and Johnson & Johnson at death prevention did not appear counteract the negative association between providing information on their overall efficacy and perceived effectiveness or intention to vaccinate. Both pieces of information simply canceled each other out. This suggests that there is a need to focus communication strategies on this metric of performance rather than the arguably less important indicator of overall effectiveness at preventing symptomatic COVID-19.

There may well be other ways to tailor this type of message for greater effectiveness. Rather than speaking of vaccine effectiveness in terms of percentages, science communicators can use plain language or graphical images to convey the effectiveness of the AstraZeneca and Johnson & Johnson vaccines. It is also possible that neutralizing information—such as explaining the substantive limitations of overall efficacy information—may counteract the effects of this information when provided in isolation, because people may not fully understand what information this metric is conveying to them. Of course, individuals can also be persuaded to vaccinate with messages that are not based on scientific data, such as appeals to norms and values or compelling narratives from individuals who have received AstraZeneca or Johnson & Johnson that account for why they did so.

Researchers can also experiment with different sources to convey these messages. Skepticism toward AstraZeneca and Johnson & Johnson has resulted from highly flawed communication of rare adverse effects through the news media. Scientists, doctors, and medical professionals are highly credible sources who could potentially enhance the persuasive potential of messages aimed at conveying the benefits of these vaccines. We encourage future research on alternative communication strategies that can enhance uptake of less-preferred COVID-19 vaccines.

### Limitations

There are a few important limitations to the data and design we present here. First, this study was conducted on an online nonprobability sample of Canadian adults. We cannot make strong claims to population generalizability. Nonetheless, the demographic characteristics of our sample closely match important population benchmarks (see eTable 2 in the Supplement), and observed treatment effects do not usually differ markedly between probability and nonprobability samples.^19^ Second, Canada is characterized by a unique dynamic where multiple COVID-19 vaccines were approved and procured with uneven access to each of them throughout the population. Other countries have different vaccines procured in varying relative quantities and have different rollout strategies. Seeing how these results travel cross-nationally is important, especially to contexts that are reliant on the AstraZeneca and Johnson & Johnson vaccines.

Third, it is worth noting that surveys can only indirectly shed light on behavior through self-reported measures. Whether providing information about the death prevention potential of AstraZeneca and Johnson & Johnson actually improves uptake of these vaccines in the real world is a nearly impossible question to address with survey research. Fourth, it is worth noting that the interventions tested here are based on metrics from trial data featuring the original strain of COVID-19. The rise of the variants has changed the efficacy profile of the COVID-19 vaccines, and more data are now available on their real-world effectiveness. Effectively communicating information about the safety and efficacy profiles of different vaccines during rapidly evolving conditions is an enormous challenge.

## Conclusions

By use of a between-participants experiment included in a survey of Canadian adults randomly assigned vaccine brand conditions, we found that providing information on the effectiveness of less-preferred vaccines like AstraZeneca and Johnson & Johnson at preventing death from COVID-19 was associated with more confidence in the effectiveness of these vaccines and more willingness to take them if offered. Information on the overall effectiveness of these vaccines at preventing symptomatic COVID-19 infection showed the opposite result. Providing information related to death prevention ability of these vaccines did not, however, mitigate the negative association between the provision of overall efficacy information and perceptions of their effectiveness or willingness to take them if offered. These results can inform public health communication strategies to reduce hesitancy toward specific COVID-19 vaccines.

## Notes

Supplement. **eAppendix.** Supplemental Results **eFigure 1.** Mean Vaccine Intention (Left) and Perceived Effectiveness (Right) Across Treatment Conditions for Preregistered Pilot Study **eFigure 2.** Mean Vaccine Intention (Left) and Perceived Effectiveness (Right) Across Treatment Conditions for Respondents in AstraZeneca Condition (Top) and Johnson & Johnson Condition (Bottom) **eTable 1.** Balance Tests **eTable 2.** Sample Characteristics **eTable 3.** OLS Estimates **eTable 4.** Ordered Logit Estimates **eFigure 3.** Ordered Logistic Regression Results **eFigure 4.** Predicted Vaccine Intention in Death Prevention Treatment and Control Conditions by Age Group (Top Panels) and Gender (Bottom Panels) for Those in the Johnson & Johnson (Left Panels) and AstraZeneca Conditions (Right Panels)
