# Supplementary material for: Assessment of Communication Strategies for Mitigating COVID-19 Vaccine-Specific Hesitancy in Canada
Source: JAMA Netw Open. 2021 Sep 30;4(9):e2126635. doi: 10.1001/jamanetworkopen.2021.26635 (PMC8485173; doi:10.1001/jamanetworkopen.2021.26635)
Supplement: Supplement. — eAppendix. Supplemental Results eFigure 1. Mean Vaccine Intention (Left) and Perceived Effectiveness (Right) Across Treatment Conditions for Preregistered Pilot Study eFigure 2. Mean Vaccine Intention (Left) and Perceived Effectiveness (Right) Across Treatment Conditions for Respondents in AstraZeneca Condition (Top) and Johnson & Johnson Condition (Bottom) eTable 1. Balance Tests eTable 2. Sample Characteristics eTable 3. OLS Estimates eTable 4. Ordered Logit Estimates eFigure 3. Ordered Logistic Regression Results eFigure 4. Predicted Vaccine Intention in Death Prevention Treatment and Control Conditions by Age Group (Top Panels) and Gender (Bottom Panels) for Those in the Johnson & Johnson (Left Panels) and AstraZeneca Conditions (Right Panels) [file jamanetwopen-e2126635-s001.pdf]

Supplemental Online Content

Merkley E, Loewen PJ. Assessment of communication strategies for mitigating COVID-19 vaccine-specific hesitancy in Canada. *JAMA Netw Open*. 2021;4(9):e2126635. doi:10.1001/jamanetworkopen.2021.26635

eAppendix. Supplemental Results

eFigure 1. Mean Vaccine Intention (Left) and Perceived Effectiveness (Right) Across Treatment Conditions for Preregistered Pilot Study

eFigure 2. Mean Vaccine Intention (Left) and Perceived Effectiveness (Right) Across Treatment Conditions for Respondents in AstraZeneca Condition (Top) and Johnson & Johnson Condition (Bottom)

eTable 1. Balance Tests

eTable 2. Sample Characteristics

eTable 3. OLS Estimates

eTable 4. Ordered Logit Estimates

eFigure 3. Ordered Logistic Regression Results

eFigure 4. Predicted Vaccine Intention in Death Prevention Treatment and Control Conditions by Age Group (Top Panels) and Gender (Bottom Panels) for Those in the Johnson & Johnson (Left Panels) and AstraZeneca Conditions (Right Panels)

This supplemental material has been provided by the authors to give readers additional information about their work.

© 2021 Merkley E et al. *JAMA Network Open.*

eAppendix. Supplemental Results

Pilot

Our pre-registered pilot study was fielded from March 8-17 2021 on a sample of 2,500 Canadian citizens 18 years or older using the online panel provider Dynata. The details of the data collection are otherwise identical to the study presented in the main text. Respondents were first randomized into four groups. The control group was given the following prime that contained no brand information about their potential COVID-19 vaccine:

Health Canada has recently approved the distribution of several COVID-19 vaccines.

The remaining groups received brand information with half receiving primes related to AstraZeneca and the other half receiving Johnson & Johnson. One of these groups received information about the brand of their vaccine and its overall effectiveness at preventing cases of symptomatic COVID-19:

Health Canada has recently approved the [AstraZeneca/Johnson & Johnson] COVID-19 vaccine for distribution. Studies have shown that the vaccine is [62%/72%] effective at preventing symptomatic COVID-19.

Another group received information that the vaccines are virtually 100% effective at preventing death from COVID-19.

Health Canada has recently approved the [AstraZeneca/Johnson & Johnson] COVID-19 vaccine for distribution. Studies have shown that the vaccine is nearly 100% effective at preventing death from COVID-19.

A final group received both pieces of information. All respondents were asked to report the likelihood of accepting a vaccine if offered (response categories: very likely, somewhat likely, not very likely, not at all likely), and their effectiveness (response categories: very effective, somewhat effective, not very effective, not at all effective).

We pre-registered expectations that respondents in the brand conditions should report lower vaccine intention and perceived efficacy compared to the control. Intention and efficacy should be higher in the death prevention condition compared to the overall efficacy condition, and they should be higher in the condition providing both pieces of information than in the condition providing information on overall efficacy alone.

The group means are displayed in Supplementary Figure 1. Consistent with the findings in the main text, intention and perceived effectiveness was 0.11 (95% CI = (-0.14, -0.08), $p < 0.001$) and 0.07 points (95% CI = (-0.09, -0.04), $p < 0.001$) lower in the brand conditions compared to the control.

Providing information about death prevention appears to matter to some degree. Intention was 0.07 points higher in the death prevention condition compared to the overall efficacy condition (95% CI = (-0.10,-0.03), $p < 0.001$), while perceived effectiveness was 0.09 points higher (95% CI = (-0.12,-0.06), $p < 0.001$). In fact, the group mean on perceived effectiveness for those in the death prevention condition converges with the control condition (though not for intention).

© 2021 Merkley E et al. *JAMA Network Open.*

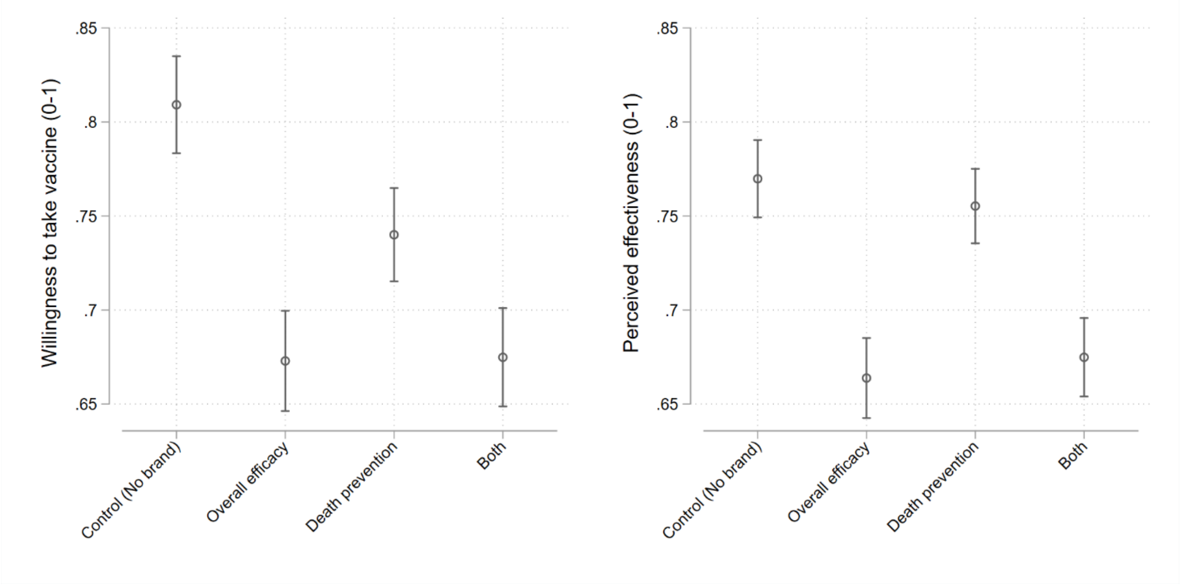

eFigure 1. Mean Vaccine Intention (Left) and Perceived Effectiveness (Right) Across Treatment Conditions for Preregistered Pilot Study. Note: 95% confidence interval. Survey fielded from March 8-17, 2021.

However, death prevention information does not appear to mitigate the effect of overall efficacy information. Respondents given both pieces of information did not report greater willingness to vaccinate (b = 0.00, 95% CI= (-0.04, 0.04), p = n.s.) or higher levels of perceived effectiveness (b = 0.01, 95% CI = (-0.02, 0.04), p = n.s.) compared to those who only received information on overall vaccine efficacy.

© 2021 Merkley E et al. *JAMA Network Open.*

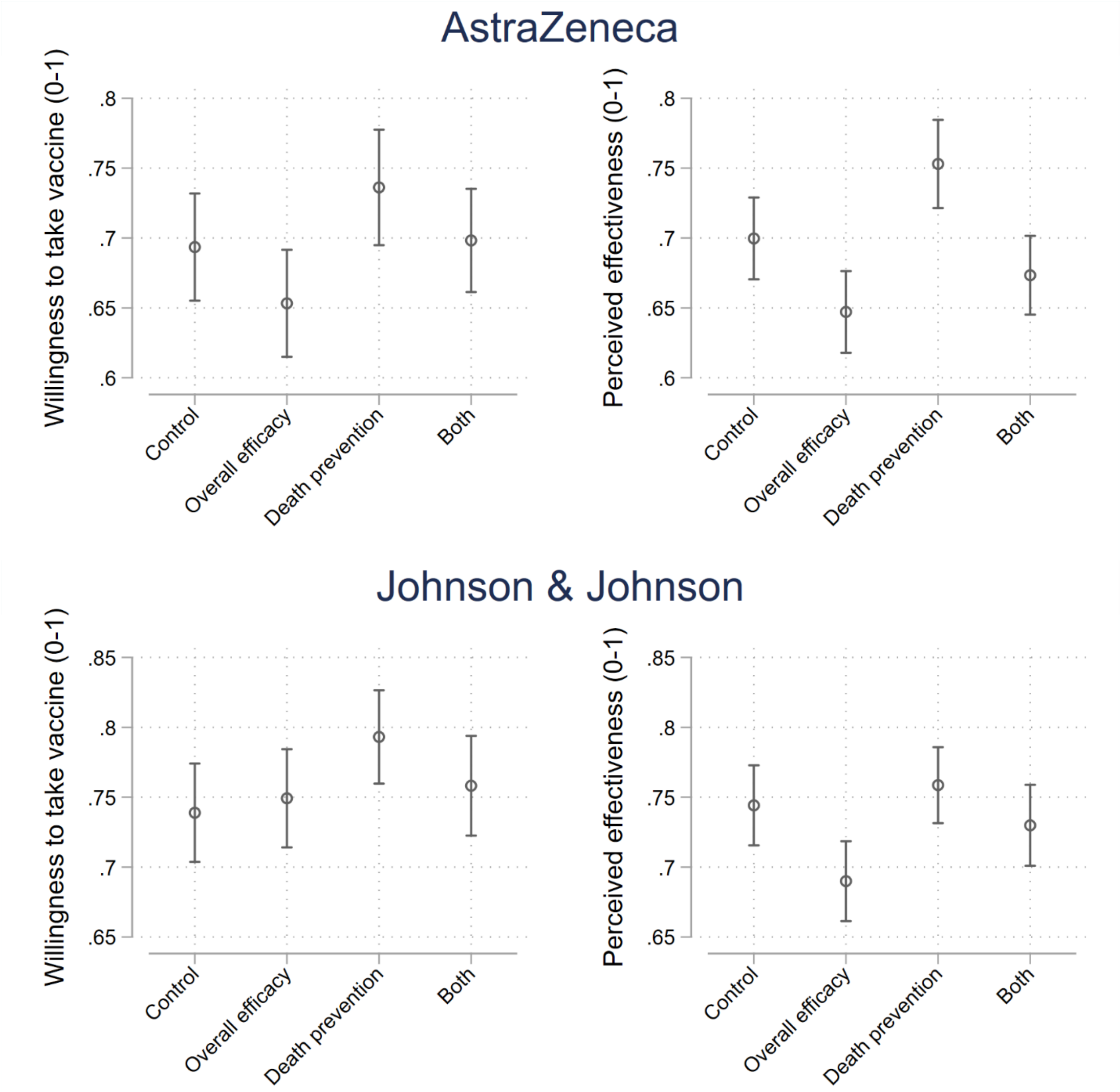

eFigure 2. Mean Vaccine Intention (Left) and Perceived Effectiveness (Right) Across Treatment Conditions for Respondents in AstraZeneca Condition (Top) and Johnson & Johnson Condition (Bottom). Note: 95% confidence intervals. Survey fielded from March 24-30, 2021.

© 2021 Merkley E et al. *JAMA Network Open.*

eTable 1. Balance Tests

Base = Control	Overall efficacy		Death prevention		Both	
	b/p	lb/ub	b/p	lb/ub	b/p	lb/ub
Education	-0.02	-0.08	0.00	-0.06	0.02	-0.05
	(0.568)	0.05	(0.879)	0.07	(0.604)	0.08
Age	-0.00	-0.01	0.00	-0.01	0.01	-0.00
	(0.626)	0.01	(0.455)	0.01	(0.116)	0.01
Income	-0.01	-0.09	0.01	-0.07	0.03	-0.05
	(0.837)	0.07	(0.852)	0.09	(0.418)	0.11
Female	-0.00	-0.26	0.06	-0.20	0.01	-0.24
	(0.987)	0.26	(0.670)	0.31	(0.941)	0.26
Quebec	0.12	-0.42	-0.14	-0.67	0.10	-0.44
	(0.662)	0.66	(0.614)	0.40	(0.712)	0.65
Ontario	-0.10	-0.61	-0.11	-0.61	0.08	-0.43
	(0.688)	0.40	(0.651)	0.38	(0.744)	0.60
West	0.20	-0.32	0.09	-0.42	0.37	-0.15
	(0.447)	0.72	(0.721)	0.60	(0.158)	0.90
Vaccination support	-0.36	-1.00	-0.11	-0.74	0.13	-0.48
	(0.268)	0.28	(0.738)	0.52	(0.669)	0.75
Ideology	0.00	-0.05	0.01	-0.05	-0.02	-0.08
	(0.873)	0.06	(0.777)	0.07	(0.402)	0.03
Science literacy	0.02	-0.06	-0.04	-0.12	-0.03	-0.11
	(0.680)	0.10	(0.349)	0.04	(0.380)	0.04
Constant	0.91	-0.92	0.15	-1.67	-0.66	-2.44
N			2027			
LR chi2			19.90			
Prob > chi2			0.92			

Note: b= coefficient estimate; p=p-value (in parentheses); lb=lower bound 95% interval; ub=upper bound 95% interval; Vaccination support measured with 0-1 index based on agreement with following: 1) Childhood vaccines are important for the health of children; 2) Getting vaccines is a good way to protect children from disease; 3) Having children vaccinated is important for the health of others in my community; 4) All childhood vaccines offered by the government in my community are beneficial; 5) The information I receive about vaccines from the government is reliable and trustworthy; 6) Generally I do what my doctor or health care provider recommends about vaccines. Ideology measured with 0-10 left-right self-placement scale. Science literacy measured with 0-1 index of the follow items: 1) The center of the Earth is very hot (True); 2) The continents have been moving their location for millions of years and will continue to move (True); 3) All radio-activity is man-made (False); 4) Electrons are smaller than atoms (True); 5) Lasers work by focusing sound waves (False); 6) It is the father's gene that decides whether the baby is a boy or a girl (True); 7) Antibiotics kill viruses as well as bacteria (False).

© 2021 Merkley E et al. *JAMA Network Open.*

eTable 2. Sample Characteristics

		% of Sample	% of Population
Female		52.4	51.4
Age	18-34	25.2	27.3
	35-54	35.0	34.1
	55+	39.9	38.6
Region	Atlantic	6.6	6.8
	Quebec	23.0	23.4
	Ontario	38.7	38.3
	West	31.6	31.2
French		20.2	21.4

Note: population benchmarks based on 2016 Canadian Census

eTable 3. OLS Estimates

	**Intention**				**Perceived Effectiveness**			
	b/p	lb/ub	b/p	lb/ub	b/p	lb/ub	b/p	lb/ub
Death prevention	0.04	0.01	0.05	0.01	0.03	0.01	0.03	0.01
	(0.004)	0.06	(0.006)	0.09	(0.002)	0.05	(0.020)	0.06
Overall efficacy	-0.03	-0.05	-0.02	-0.05	-0.05	-0.08	-0.05	-0.08
	(0.033)	-0.00	(0.417)	0.02	(0.000)	-0.03	(0.000)	-0.02
Overall * death prevention			-0.03	-0.08			-0.00	-0.04
			(0.324)	0.03			(0.888)	0.04
Constant	0.72	0.70	0.72	0.69	0.72	0.70	0.72	0.70
	(0.000)	0.75	(0.000)	0.74	(0.000)	0.74	(0.000)	0.74
R^2	0.01		0.01		0.01		0.01	
N	2556		2556		2556		2556	

Note: b= coefficient estimate; p=p-value (in parentheses); lb=lower bound 95% interval; ub=upper bound 95% interval

© 2021 Merkley E et al. *JAMA Network Open.*

eTable 4. Ordered Logit Estimates

	Intention				Perceived Effectiveness			
	b/p	lb/ub	b/p	lb/ub	b/p	lb/ub	b/p	lb/ub
Death prevention	0.22	0.08	0.34	0.13	0.26	0.11	0.32	0.11
	(0.003)	0.37	(0.001)	0.56	(0.001)	0.41	(0.003)	0.53
Overall efficacy	-0.17	-0.32	-0.06	-0.26	-0.45	-0.60	-0.39	-0.59
	(0.023)	-0.02	(0.597)	0.15	(0.000)	-0.30	(0.000)	-0.18
Overall * death prevention			-0.23	-0.53			-0.13	-0.42
			(0.117)	0.06			(0.403)	0.17
Cut point 1	-2.12	-2.28	-2.06	-2.24	-3.08	-3.29	-3.05	-3.27
	(0.000)	-1.96	(0.000)	-1.88	(0.000)	-2.87	(0.000)	-2.83
Cut point 2	-1.22	-1.35	-1.16	-1.31	-1.78	-1.93	-1.75	-1.91
	(0.000)	-1.08	(0.000)	-1.00	(0.000)	-1.63	(0.000)	-1.58
Cut point 3	-0.03	-0.15	0.03	-0.11	0.56	0.43	0.59	0.44
	(0.702)	0.10	(0.668)	0.18	(0.000)	0.69	(0.000)	0.74
N	2556		2556		2556		2556	

Note: b= coefficient estimate; p=p-value (in parentheses); lb=lower bound 95% interval; ub=upper bound 95% interval

© 2021 Merkley E et al. *JAMA Network Open.*

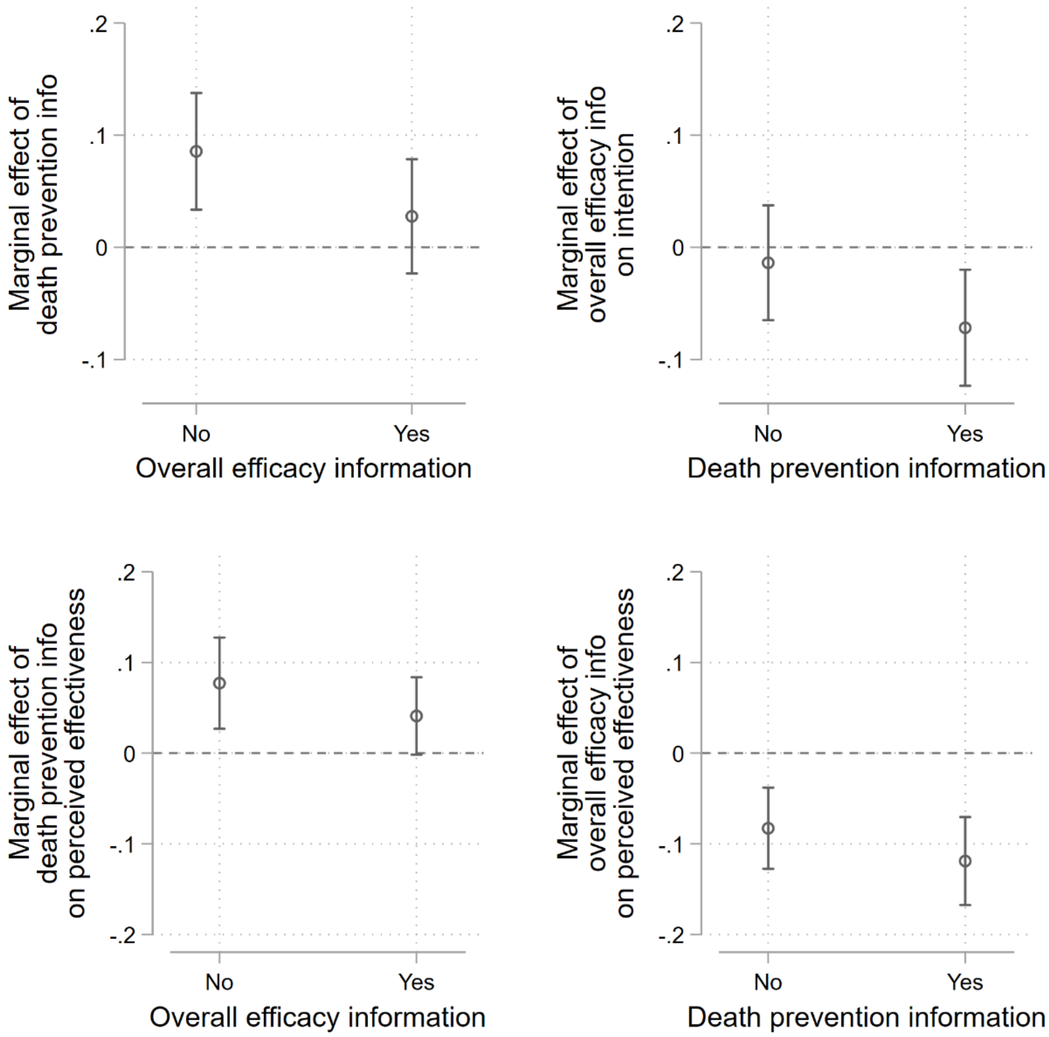

eFigure 3. Ordered Logistic Regression Results. Marginal effect of death prevention information by overall efficacy condition on vaccine intention (top-left) and perceived effectiveness (bottom-left). Marginal effect of overall efficacy information by death prevention condition on vaccine intention (top-right) and perceived effectiveness (bottom-right). Note: 95% confidence intervals. Regression estimates found in Supplementary Table 4. Effects are expressed as increases or decreases in the outcome equally "very likely" or "very effective".

© 2021 Merkley E et al. *JAMA Network Open.*

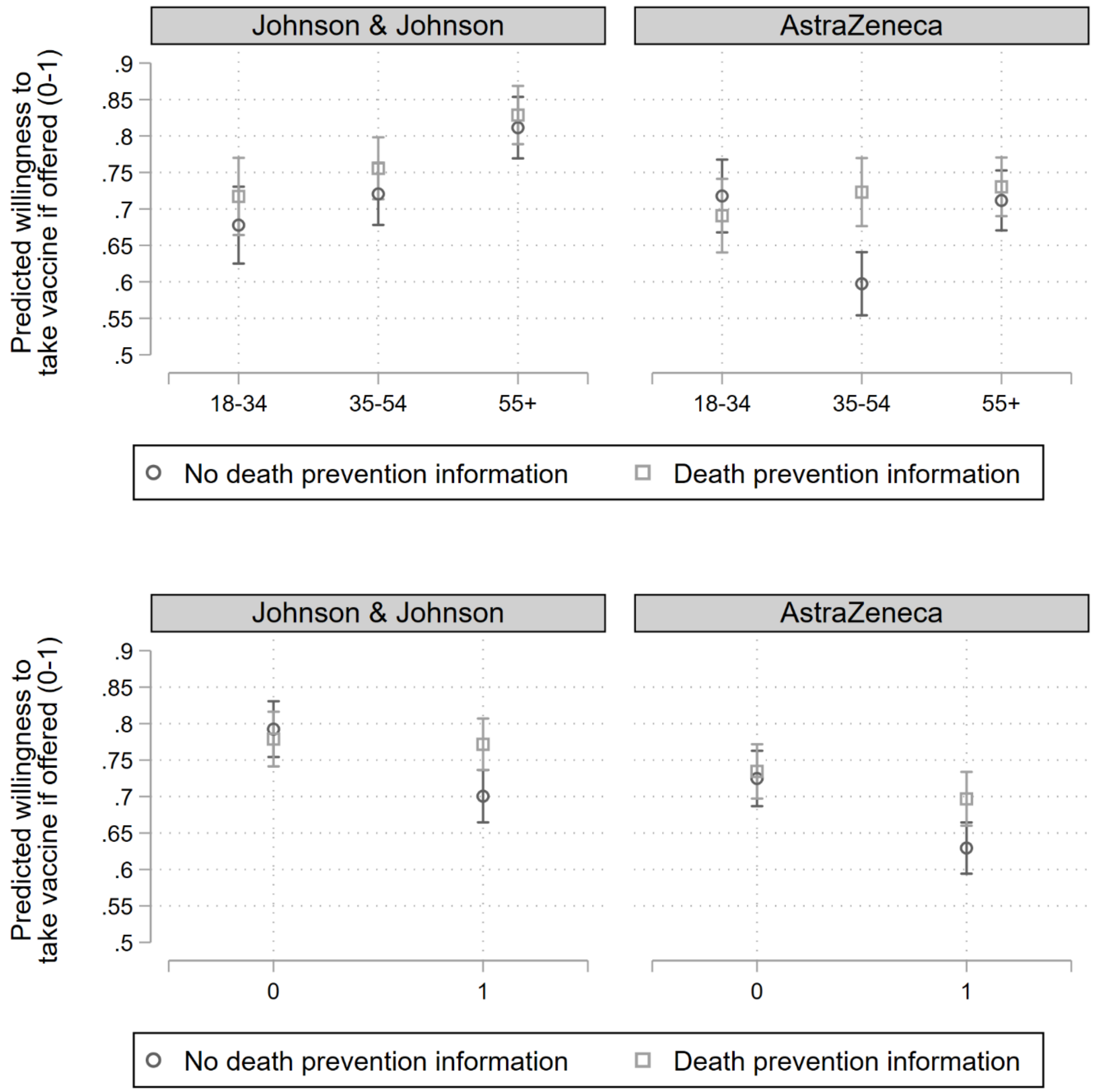

eFigure 4. Predicted Vaccine Intention in Death Prevention Treatment and Control Conditions by Age Group (Top Panels) and Gender (Bottom Panels) for Those in the Johnson & Johnson (Left Panels) and AstraZeneca Conditions (Right Panels). Note: 95% confidence intervals. Regression estimates found in Tables 1 and 2. Survey fielded from March 24-30, 2021.

© 2021 Merkley E et al. *JAMA Network Open.*
